# Research Note: Impact of applied thermal treatment on textural, and sensory properties and cooking loss of selected chicken and turkey cuts as affected by cooking technique

**DOI:** 10.1016/j.psj.2022.101923

**Published:** 2022-04-22

**Authors:** Robert Gál, Josef Kameník, Richardos Nikolaos Salek, Zdeněk Polášek, Blanka Macharáčková, Tomáš Valenta, Danka Haruštiaková, Štěpán Vinter

**Affiliations:** ⁎ Department of Food Technology, Faculty of Technology, Tomas Bata University in Zlín, Zlín 760 01, Czechia; † University of Veterinary Sciences Brno, Faculty of Veterinary Hygiene and Ecology, Brno 612 42, Czechia; ǂ Masaryk University, Faculty of Science, RECETOX, Brno 602 00, Czechia; § Masaryk University, Faculty of Medicine, Institute of Biostatistics and Analyses, Brno 625 00, Czechia; # Department of Environmental Protection Engineering, Faculty of Technology, Tomas Bata University in Zlín, Zlín 760 01, Czechia

**Keywords:** cooking, poultry meat, cooking loss, shear force, sensory properties

## Abstract

The effect of various cooking methods (roasting, broiling, grilling, frying, and stewing) on cooking loss (**CL**) and textural and sensory properties of selected chicken (breast fillet, thigh, and thigh fillet) and turkey (breast fillet, thigh) cuts in relation to the applied apparatus was evaluated. Diverse results were recorded according to the method, the type of poultry meat, and the cut of poultry meat. Additionally, CL and shear force (**SF**) values in all examined samples were influenced by the culinary technique, the type of poultry meat, and the poultry meat cut. The lowest CL and shear SF values were reported when the samples were treated using a method with higher heating rates and/or temperatures and shorter cooking times. Additionally, lower values of CL and SF were obtained for chicken meat compared to turkey meat (thighs). In general, the applied culinary technique affected the sensory properties of the samples tested. High sensory scores were recorded for grilled chicken breast fillets and fried turkey breast fillets (irrespective of the applied apparatus). On the whole, it could be stated that culinary techniques at high temperature requiring shorter times (such as frying, grilling, and roasting) were evaluated to be more effective (in terms of CL and SF).

## INTRODUCTION

The production and consumption of poultry meat has increased in many parts of the world in recent decades. A large group of consumers include poultry meat (and products thereof) in their dietary habits due to the fact that the abovementioned products contain a higher protein level and a lower fat content than other meats. Hence, poultry meat is convenient, satisfies consumer preferences, is affordable, and does not face religious restrictions. Furthermore, chicken and turkey meat are the most consumed meat types (among poultry) because of their high nutritional value, ease of cooking and digestion, and economic feasibility (Park et al., 2020).

The cooking (thermal treatment/culinary technique) of meat plays a key role in achieving palatable and safe consumption of the final product. Moreover, the culinary technique utilized affects a wide range of meat quality and sensory attributes. In particular, structural changes may occur due to protein denaturation.

The texture of cooked meat is generally associated with heat-induced changes in connective tissue, soluble proteins, and myofibrillar proteins. Moreover, the extent of cooking loss (**CL**) directly influences both tenderness and juiciness. CL occurs as a result of the evaporation of water and the dripping of water and oil. Therefore, the culinary technique utilized depends on the extent of acceptable CL (Murphy and Marks, 2000).

Modern lifestyles promote the consumption of food outside the home, highlighting the importance of the modern food industry (including the food service, catering service, and restaurant sectors) (Macharáčková et al., 2021; Ortuño et al., 2021; Sruthi et al., 2021). Therefore, the aim of the study was to evaluate the effects of various culinary techniques performed on different apparatuses on CL, Warner–Bratzler shear force (**SF**; tenderness), and sensory attributes of different cuts of chicken and turkey meat.

## MATERIALS AND METHODS

### Materials

Samples of poultry meat (*Gallus domesticus*, female Ross 308, 35 d of age, ca 1.3 kg, 24 h postmortem, N = 240; *Meleagris gallopavo f. domestica*, female BUT Big 6, 295 d of age, ca 7.1 kg, 24 h postmortem, N = 160) were cooked and analyzed. Birds were housed in floor pens and reared under controlled environmental conditions (fully complied with standards for the fattening of Ross 308 chickens and BUT Big 6 turkeys). A 3-phase and 4-phase feeding program were used for fattened chickens and turkeys, respectively. The diets used contained recommended nutrients composition according to management guidelines for growing Ross 308 chickens and BUT turkeys. Chicken and turkey meat was obtained from the local market. Two different cuts (breast and thigh) were used for each type of poultry meat. The samples were stored under refrigeration conditions (2 ± 2°C) until thermal treatment was performed (24 h after purchase). All analyses were performed 24 h after cooking.

### Thermal Treatment Methods

Broiling, grilling, frying, stewing (chicken breast fillet: **CBF**); roasting (chicken thigh: **CT**); broiling, grilling (chicken thigh fillet: **CTF**); broiling, grilling, frying (turkey breast fillet: **TBF**); and roasting (turkey thigh: **TT**) were applied. All samples were prepared at 1.00% w/w NaCl concentrations (surface salting) then placed in polyethylene bags and vacuum-packed and stored (2 ± 2°C for 6 h). The total cooking time for all methods was in the range of 7 to 16 min until the center of the product reached 75 ± 1°C (controlled by a thermometer probe directly into the sample). The roasting/broiling process was conducted using a combi-oven (**CO**; convection air steam; SelfCookingCenter, SCC WE 61; RATIONAL Czech Republic s.r.o., Prague, Czechia; operating at 163°C and 35 to 55% rel. humidity), a multifunctional electric pan (**MEP**; VarioCookingCenter 112T; RATIONAL Czech Republic s.r.o., Prague, Czechia; cooking surface: 12 dm^2^; cooking volume: 14 L), and a hot air oven (**HAO**; forced air convection; Gorenje BO758A47XG; Prague, Czechia; operating at 163°C). Additionally, samples were grilled using a double-sided contact grill (**CG**; De'Longhi CGH 1030 D, De'Longhi Praga s.r.o., Prague, Czechia), in which the temperature was set at 163°C and a commercial outdoor gas grill (**GG**; Campingaz 2 SERIES Classic L, CAMPING GAZ CS s.r.o., Prague, Czechia). The frying process was performed in a CO and on a non-stick pan (**PF**; bottom diameter: 280 mm; Tefal Unlimited G2550672; Groupe SEB ČR s.r.o., Prague, Czechia). Before frying the samples were coated with wheat flour (Goodmills Česko s.r.o., Kyjov, Czechia), dipped in a beaten whole egg (Drůbežárna Holešov s r.o., Holešov, Czechia) and then coated with breadcrumbs (PENAM, a.s., Brno, Czechia). Only one sample was fried in each batch of frying (the samples were turned twice during frying) in order to reduce the alteration of temperature. After each batch, the oil used was replaced, and any excess oil from the sample was removed using tissue paper. Furthermore, the stewing process was conducted in a convection air CO and a MEP and water (750–800 mL per 1 kg of sample) was used as liquid medium.

### Laboratory Analyses

Raw poultry meat samples were analyzed for moisture, total protein and total lipid contents (AOAC, 2000). Non-collagen muscle protein (**NCMP**), collagen contents and pH values were determined according to Polášek et al. (2021). All analyses were performed at least in triplicate. The NaCl content of the samples (after thermal treatment) was determined using atomic absorption spectrometry according to Macharáčková et al. (2021) with slight modifications.

CL values were determined according to Barbanti and Pasquini (2005) and expressed as g/100 g by weight difference between thermally untreated and thermally treated samples.

Warner–Bratzler SF test was performed using a texture analyzer (TA.XT.plus; Stable Micro Systems Ltd., Godalming, UK) according to Polášek et al. (2021). The SF (N) was determined as the maximum force representing the maximum resistance of the sample to the cut.

### Sensory Analysis

Sensory analysis was carried out by a panel of 12 selected assessors (experts; 19 and 57 years of age) consuming poultry meat regularly. Samples were served on white plates, odor-free, and covered with aluminum foil (in random order and at a controlled temperature of 60 ± 1 °C) in a sensory laboratory (under normal light conditions). Water was provided for mouth-rinsing between the samples to avoid carry-over effects. The sensory descriptors evaluated were: appearance on the surface, color on the cut surface, aroma pleasantness, juiciness, tenderness, disintegration of muscle bundles, saltiness, flavor, metallic taste, and overall impression.

### Statistical Analysis

Data were analyzed using one-way ANOVA followed by Tukey's post hoc test (basic chemical analysis) and factorial ANOVA followed by Tukey's post hoc test (the effect of the culinary technique and origin and cut of the poultry meat on CL and SF). Results were expressed as mean ± SEM. Statistical significance was set at *P* < 0.05. The multivariate method of principal component analysis (**PCA**) was used to assess the relationships between CL, SF, NaCl, and sensory properties. Statistica ver. 13 (TIBCO Software Inc., CA) software was used for the analyses.

## RESULTS AND DISCUSSION

The TBF had the highest moisture (75.45 ± 0.12% w/w) and protein (20.76 ± 0.25% w/w) content, while having a low-fat (1.85 ± 0.03% w/w) content; CBF exhibited similar values for moisture (74.14 ± 0.19% w/w), protein (20.86 ± 0.93% w/w), and fat (1.33 ± 0.01% w/w) content. A notably higher fat level was observed for CT (whole; 5.31 ± 0.04% w/w), CTF (5.39 ± 0.05% w/w), and TT (4.91 ± 0.04% w/w). The NCMP content of the raw samples was in the range of 16.96 to 20.73% w/w and the collagen content from 0.18 to 0.69% w/w. The pH values of the raw samples were in the range of 5.99 to 6.46. The composition of the raw samples was similar to those reported by other researchers (Barbanti and Pasquini, 2005). In addition, NaCl content (mg/kg) results for all tested samples (regardless of the cooking technique and equipment used) were the following: chicken breast (fillet: 11,798.1–12,559.5; cubes: 11,241.7–12,795.5), chicken thigh (fillet: 14,818.7–15,149.7; whole: 13,449.7–15,875.6), turkey breast (fillet: 14,658.1–15,702.8; cubes: 13,174.4–15,875.9), and turkey thigh (fillet: 13,584.7–14,267.1; whole: 14,217.7–15,024.75).

CL in chicken and turkey breasts and thigh were influenced by the culinary technique, type of poultry meat, type of cut, and their interaction (factorial ANOVA, effect of culinary technique: F(11,471) = 24.412, *P* < 0.001; effect of chicken/turkey: F(1,471) = 5.716, *P* = 0.017; effect of breast/thigh: F(1,471) = 13.654, *P* < 0.001; effect of culinary technique and meat types interaction: F(11,471) = 6.997, *P* < 0.001). CBF showed an average CL of 18.79% (stewing in MEP) to 26.83% (broiling in MEP). The average CL of the chicken cubes prepared by broiling ranged from 26.28% (CO) to 32.04% (MEP). In addition, when focusing on the same culinary technique, no differences in CL in CBF were found using different apparatuses. For TBF, the highest CL was for samples prepared by broiling in MEP (30.30%) and the lowest in samples prepared by panfrying (11.98%). CL of 21.76% (broiling, CO) and 26.75% (broiling, MEP) were observed in the turkey cubes. Significant differences in CL between chicken and turkey breasts were found only for the panfrying technique (Table 1). CL in the thighs differed significantly between culinary techniques as well as between the type of poultry meat. In particular, when roasting the whole thigh, higher CL was found in samples prepared in the CO than that in the HAO, both in chicken (CO 26.30%; HAO 13.63%) and turkey (CO 36.85%; HAO 20.23%) meat. Furthermore, there was a significant difference in CL between chicken and turkey thigh samples roasted in the CO. Regarding CTF, lower CL were reported during broiling in CO (18.41%) and grilling (15.67%), and higher when broiling in MEP (28.04%). When comparing CBF and CTF prepared by the same culinary technique, differences in CL were found only for grilling treatment (Table 1). Lower CL could be attributed to the short cooking time, or it could be the result of a more defined crust formation at the surface of the sample physically trapping water in the interior of the product (Vittadini et al., 2005). Moreover, CL can be explained mainly by the evaporation of water and loss of fat during cooking. Culinary techniques requiring longer times to reach the final target temperature do not require “shock” proteins, thus limiting the amount of CL. Different rates of protein denaturation occur when divergent culinary techniques are utilized (due to different rates or extent of thermal treatment), thus leading to different time courses of structural change in poultry meat. CL is a parameter affecting the appearance of a product. Hence, a higher CL value provides the expectation of a less-optimal eating quality (Aaslyng et al., 2003). The results of our study are in accordance with the abovementioned statement.

**Table 1:** Cooking loss (%) and shear force (N) following the cooking of chicken and turkey meat.

	Culinary technique			Cooking loss (%)		Cooking loss (%)		Shear force (N)		Shear force (N)	
	Cut	Technique	Device*	Chicken meat		Turkey meat		Chicken meat		Turkey meat	
				N	mean ± st.error	N	mean ± st.error	N	mean ± st.error	N	mean ± st.error
Breast	Fillet⁎⁎	Broiling	CO	16	23S.08 ± 0.43 AB^,^^a^^,^^A^	16	20.10 ± 1.01 AB^,^^a^	117	15.66 ± 0.49 A^,^^a^^,^^A^	128	15.01 ± 0.34 A^,^^a^
	Fillet	Broiling	MEP	32	26.83 ± 2.44 BC^,^^a^^,^*^A^*	32	30.30 ± 1.31 C^,^^a^	128	8.87 ± 0.23 A^,^^a^^.^^A^	128	16.44 ± 0.84 A^,^^a^
	Fillet	Grilling	GG	16	23.66 ± 0.70 AB^,^^a^	16	22.40 ± 1.16 BC^,^^a^	128	13.52 ± 0.43 A^,^^a^	128	11.64 ± 0.42 A^,^^a^
	Fillet	Grilling	CG	16	25.83 ± 1.50 ABC^,^^a^^,^^A^	16	19.58 ± 1.52 AB^,^^a^	128	9.16 ± 0.24 A^,^^a^^.^^A^	128	17.08 ± 0.44 A^,^^a^
	Fillet	Frying⁎⁎	CO	16	18.92 ± 0.47 AB^,^^a^	16	12.09 ± 0.95 A^,^^a^	128	9.75 ± 0.32^A^^,^^a^	128	12.58 ± 0.33 A^,^^a^
	Fillet	Frying	PF	16	23.57 ± 0.68 AB^,^^a^	16	11.98 ± 4.94 A^,^^b^	128	10.06 ± 0.28 A^,^^a^	128	11.69 ± 0.32 A^,^^a^
	Fillet	Stewing	CO	16	19.71 ± 0.32 AB			128	11.16 ± 0.37 A^,^^a^		
	Fillet	Stewing	MEP	32	18.79 ± 0.48 A			128	7.53 ± 0.25 A^,^^a^		
	Cubes	Broiling	MEP	32	32.04 ± 0.49 C^,^^a^	32	26.75 ± 1.48 BC^,^^a^	128	6.80 ± 0.23 A^,^^a^	123	12.59 ± 0.30 A^,^^a^
	Cubes	Broiling	CO	16	26.28 ± 0.87 ABC^,^^a^	16	21.76 ± 1.18 B^,^^a^	128	13.15 ± 0.31 A^,^^a^	128	78.35 ± 9.49 B^,^^b^
Thigh	Whole	Roasting	HAO	16	13.63 ± 0.63 A^,^^a^	16	20.23 ± 1.66 A^,^^a^	128	12.29 ± 0.37 A^,^^a^	128	27.67 ± 1.19 A^,^^b^
	Whole	Roasting	CO	16	26.30 ± 0.59 BC^,^^a^	16	36.85 ± 1.78 B^,^^b^	128	14.80 ± 0.46 A^,^^a^	128	18.16 ± 0.69 A^,^^b^
	Fillet	Broiling	CO	16	18.41 ± 0.73 AB^,^^A^			128	7.20 ± 0.22 A^,^^A^		
	Fillet	Broiling	MEP	32	28.04 ± 1.84 C^,^^A^			128	9.47 ± 0.27 A^,^^A^		
	Fillet	Grilling	CG	16	15.67 ± 0.63 A^,^^B^			128	8.75 ± 0.21 A^,^^A^		

A,B,C Cooking loss and shear force, respectively, in poultry meat prepared by various culinary techniques followed by the same capital letter in the column did not differ significantly (Tukey's post hoc test in factorial ANOVA; separately for breast and thigh).

a,b Cooking loss and shear force, respectively, in chicken and turkey meat prepared by the same culinary technique followed by the same lower-case letter in the row did not differ significantly (Tukey's post hoc test in factorial ANOVA).

A,B Cooking loss and shear force, respectively, in ther breast and thigh followed by the same capital letter in italics in the column in the corresponding row did not differ significantly (Tukey's post hoc test in factorial ANOVA).

⁎ CG, contact grill; CO, combi-oven; GG, gas grill; HOA, hot air oven; MEP, multifunctional electric pan; PF, pan-frying.

⁎⁎ Cooking loss was calculated without considering the weight of the crumb (after frying, the crumb was removed). Chicken breast fillet: thickness: 2.45–3.75 cm, weight: 160–200 g; Chicken thigh fillet: thickness: 2.05–2.46 cm, weight: 194–227 g; Turkey breast fillet: thickness: 2.75–3.85 cm, weight: 267–315 g; Chicken thigh: weight: 220–250 g; Turkey thigh weight: 450–470 g.

According to Barbanti and Pasquini (2005), SF can be used as an index of poultry meat tenderness. Chicken and turkey breasts SF values ranged from 6.80 N to 17.08 N for all treatments and apparatuses, except turkey breast cubes broiled in a CO, where a significantly higher value of SF (78.35 N) was obtained. SF in thighs ranged from 7.20 to 27.67 N. Lower SF values were recorded in CTF (7.20–9.47 N), and higher values in thighs of both types—chicken and turkey—roasted as a whole (12.29–27.67 N). When roasting thigh as a whole, higher SF values were reported for turkey meat, however, the difference was significant only for roasting in HAO (Table 1). Due to the latter discrepancy in the breast, the SF was affected by both the culinary technique and the type of poultry meat (chicken/turkey), but not by the type of cut (breast/thigh); the interaction of these factors was also significant (factorial ANOVA, effect of culinary technique: F(11,3159) = 48.860, *P* < 0.001; effect of chicken/turkey: F(1,3159) = 149.259, *P* < 0.001; effect of breast/thigh: F(1,3159) = 0.008, *P* = 0.929; effect of culinary technique and meat types interaction: F(11,3159) = 41.983, *P* < 0.001).

**Figure 1:**
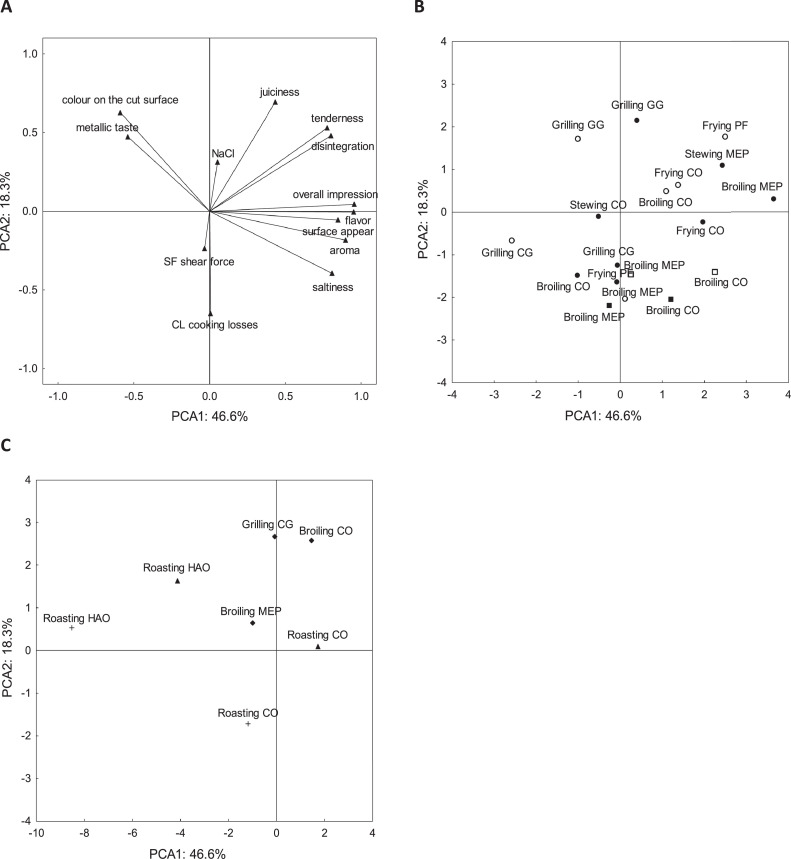
Position of all the properties measured of chicken and turkey meat in the first 2 principal components of principal component analysis (PCA; λ_1_ = 6.056, λ_2_ = 2.375) (A). The position of chicken and turkey breast samples in the first 2 principal components of PCA (B). The position of chicken and turkey thigh samples in the first 2 principal components of PCA (C). The labels of the samples indicate the cooking method followed by the abbreviation of the device: CO – Combi-oven, MEP – Multifunctional electric pan, GG – Gas grill, CG – Contact grill, PF – Pan-frying, HOA – Hot air oven. Symbols of samples indicate the type of poultry meat: ● Chicken breast fillet, ■ Chicken breast cubes, ○ Turkey breast fillet, □ Turkey breast cubes, ▲ Chicken thigh whole, ⬩ Chicken thigh fillet, + Turkey thigh whole.

According to PCA, the first 2 principal components explained 64.9% of the total variance in the data. The position of the variables in the ordination space confirmed a positive correlation between CL and SF and a strong negative correlation between CL and NaCl content and juiciness, which were positively correlated with each other. Surface appearance, flavor, pleasantness of aroma, and the overall impression were positively correlated with one another and were independent of CL, SF, and juiciness (Figure 1A). CBF stewed or broiled in MEP were characterized by high values of flavor, surface appearance, aroma, disintegration, and overall impression. Furthermore, CBF grilled on GG showed the highest tenderness and juiciness. The treatment of CBF in CO led to “good” sensory properties. Fried TBF in a pan or in a CO received the highest rating for most sensory properties. Poultry meat broiled in CO was also characterized by higher property values. However, the lowest values of most properties were reported for TBF grilled on a CG. This treatment appears to be less suitable for poultry meat. In addition, when breast cubes were broiled, higher CL values were obtained, especially when broiled in MEP (Figure 1B). In the case of CTF, broiling in CO or grilling may be the most appropriate treatments, in which low CL and high scores have been found for juiciness and tenderness. When roasting CT as a whole, higher values of most sensory properties were observed in meat roasted in CO, although CL were lower when roasting in HAO. The same situation holds true for TT, where the treatment in the CO led to “better” sensory properties than treatment in the HAO. Although CL after roasting in the HAO was lower, this treatment resulted in the worst flavor and overall impression (Figure 1C).

The quality and economic aspects of food and its production offered by the food service sector are gaining increasing attention, and CL are of great economic importance (Aaslyng et al., 2003; Ortuño et al., 2021). Prolonged cooking times and higher temperatures (≥140°C) promote Maillard reactions leading to the development of volatile organic compounds that are related to typical sensory attributes of thermally treated poultry meat. Additionally, culinary techniques that involve high temperatures in an aerobic environment for a limited time provide enhanced flavor and color development, while simultaneously preventing CL (Ortuňo et al., 2021; Sruthi et al., 2021).

## Appendix Supplementary materials

Image, application 1
