# Supplementary material for: Research Note: Impact of applied thermal treatment on textural, and sensory properties and cooking loss of selected chicken and turkey cuts as affected by cooking technique
Source: Poult Sci. 2022 Apr 22;101(7):101923. doi: 10.1016/j.psj.2022.101923 (PMC9189220; doi:10.1016/j.psj.2022.101923)
Supplement: Supplementary file 1 [file mmc1.pdf]

Certificate of Elsevier Language Editing Services

ELSEVIER

The following article was edited by Elsevier Language Editing Services:

"The impact of applied thermal treatment method on the cooking loss, textural and sensory properties of selected chicken and turkey cuts as affected by the cooking technique"

Authored by:

Robert Gál

Date: 25-Nov-2021

Serial number: LEEX-16239-05816113C923
